# The Macrophage Mannose Receptor Regulate Mannan-Induced Psoriasis, Psoriatic Arthritis, and Rheumatoid Arthritis-Like Disease Models

**DOI:** 10.3389/fimmu.2018.00114

**Published:** 2018-02-06

**Authors:** Cecilia Hagert, Outi Sareila, Tiina Kelkka, Sirpa Jalkanen, Rikard Holmdahl

**Affiliations:** ^1^Medicity Research Laboratory, University of Turku, Turku, Finland; ^2^The National Doctoral Programme in Informational and Structural Biology (ISB), Turku, Finland; ^3^Medical Inflammation Research, Karolinska Institutet, Stockholm, Sweden; ^4^The Turku Doctoral Programme of Biomedical Sciences (TuBS), Turku, Finland

**Keywords:** macrophage mannose receptor (CD206), rheumatoid arthritis, psoriatic arthritis, psoriasis, reactive oxygen species, mannan

## Abstract

The injection of mannan into mice can result in the development of psoriasis (Ps) and psoriatic arthritis (PsA), whereas co-injection with antibodies toward collagen type II leads to a chronic rheumatoid-like arthritis. The critical event in all these diseases is mannan-mediated activation of macrophages, causing more severe disease if the macrophages are deficient in neutrophil cytosolic factor 1 (Ncf1), i.e., lack the capacity to make a reactive oxygen species (ROS) burst. In this study, we investigated the role of one of the receptors binding mannan; the macrophage mannose receptor (MR, CD206). MR is a C-type lectin present on myeloid cells and lymphatics. We found that mice deficient in MR expression had more severe mannan-induced Ps, PsA as well as rheumatoid-like arthritis. Interestingly, the MR-mediated protection was partly lost in *Ncf1* mutated mice and was associated with an type 2 macrophage expansion. In conclusion, these results show that MR protects against a pathogenic inflammatory macrophage response induced by mannan and is associated with induction of ROS.

## Introduction

Rheumatoid arthritis (RA), psoriasis (Ps), and psoriatic arthritis (PsA) are common diseases in human, yet insufficiently understood. They are chronic, inflammatory, and genetically dependent diseases, induced by unknown environmental factors (1, 2). Ps is characterized by inflammation and hyperproliferation of the skin, while joints are affected in PsA. Ps affects approximately 2–3% of the population worldwide, and approximately 25% of Ps patients develop PsA. The cause of PsA is not known; however, inflammation-induced stimuli, physical injuries to the skin (the “Koebner response”), and various infections in genetically susceptible individuals have been shown to initiate or exacerbate psoriatic lesions. For instance, increased *Candida* infections have been reported in Ps patients and *Candida* have been reported as an initiator of various skin diseases (1, 3–5). The disease is strongly associated with the major histocompatibility (MHC) region, but not with class II genes as in RA but with non-classical class I genes (6). RA is present in 0.5% of the population. It causes pain and inflammatory erosions of the joints, affects the cardiovascular system, and has several other comorbidities. RA leads to disability and a decreased quality of life if left untreated. RA is in part genetically dependent; among the identified genes are specific MHC class II genes, protein tyrosine phosphatase, non-receptor type 22, and neutrophil cytosolic factor 1 (*Ncf1*) (2, 7, 8).

Mannan-induced psoriasis (MIP) is a novel mouse model for PsA (induced by a single injection of mannan into mice) causing arthritic symptoms such as swelling and redness of the paws and the characteristic lesions of Ps. Mannan activates macrophages causing a production of TNF-α, which triggers IL17A secretion from γδ T cells resulting in local Ps-like inflammation. The disease is more severe under an environment with impaired reactive oxygen species (ROS), as NOX2 derived ROS in macrophages regulate disease severity. Interestingly, the disease is independent of the adaptive immune system players such as αβ T and B cells (3, 4).

Mannan can also induce chronic arthritis mimicking the development of RA, enhanced in both incidence and severity by a concomitant injection of anti-collagen type II antibodies (9).^1^ Mannan-enhanced collagen antibody-induced arthritis (mCAIA) is characterized by its dependency of ROS deficient macrophages and the complement pathway to develop. Similar to MIP, mCAIA is also independent of the adaptive immune system (see text footnote 1), although the location and erosions levels of the inflammation, the chronicity, and the effector mechanisms differ. MIP is dependent on IL17-mediated activation of innate lymphocytes whereas mCAIA is more critically dependent on the complement system.

However, the receptors, important for the recognition and initiation of these mannan-induced diseases, are unknown. This study focused on investigating one of the receptors binding mannan, the macrophage mannose receptor (MR, also known as CD206) and its role in MIP and mCAIA (10). MR is a C-type lectin receptor present on myeloid and endothelial cells (11). It is a type I membrane receptor mediating the endocytosis of glycoproteins by macrophages. It binds high-mannose containing structures on the surface of potentially pathogenic bacteria, viruses, and fungi traditionally thought to facilitate neutralization by phagocytic engulfment (12). Furthermore, investigation of human skin biopsies showed an increase of the MR (human and mouse MR are homologs) in psoriatic and dermatitis patients compared to normal controls (13, 14), and human MR was indicated to induce IL17 production (15). Its role in the immune system is not fully clarified but it is believed to be an important scavenger receptor (16, 17) and to be involved in antigen presentation to T cells by certain macrophage and dendritic cell subsets (18–20). It is proposed to have a role in facilitating phagocytosis but the MR-deficient mice are not more infectious prone than wild-type mice (21, 22). Herein, we found that mice lacking MR develop a more severe Ps and PsA and in particular a more severe chronic rheumatoid-like arthritis, suggesting that MR has a ROS-dependent protective effect on innate immunity.

## Materials and Methods

### Mice

Age- and sex-matched 6- to 12-week-old MR-deficient mice (23) with (BQ.Ncf1^m1J^) (24) or without (BQ.) the m1J mutation in *Ncf1* gene were utilized. The mice were back-crossed more than 10 times to BQ from B6 background. WT is regarded as a B10.Q mice with normal *MR* and *Ncf1* gene, Genotyping was performed with PCR, using the primers 5′-GAC CTT GGA CTG AGC AAA GGGG-3′, 5′-AGC TCG ATG CGG TTC ACC AG-3′, 5′-CTG AGA ATC CCC GCG TCCTC-3′ to detect the presence or absence of the MR gene, adapted from Lee et al. (23). The detection method of the Ncf1 point mutation has previously been described (24).

If not otherwise noted, littermates were used in the experiments. Mice were housed under specific pathogen-free conditions as described earlier (25). The study was approved by the National Animal Experiment Board in Finland, ethical permit numbers ESAVI-0000497/041003/2011 and ESAVI/439/04.10.07/2017.

### *In Vivo* Injections

Mannan-induced psoriasis was introduced by a singular intra peritoneal injection of 10 mg mannan from *Saccharomyces cerevisiae* (Sigma #M7504). The mice were blindly assessed for both arthritic symptoms and psoriatic lesions according to standardized macroscopic scoring system (3, 5, 25).

As described previously (see text footnote 1), mannan-enhanced collagen antibody-induced arthritis (CAIA) is induced by a cocktail of four monoclonal anti-collagen type II antibodies injected i.v., followed by an i.p. injection of mannan day 5.

Type 2 macrophages (M2) were induced by an injection of 5 μg recombinant mouse interleukin 4 (rmIL4; Peprotech #214-14) and 25 μg anti-IL4 antibody (BD Biosciences #554387) in PBS (Sigma, Helsinki, Finland) on days 0 and 2, mice were then euthanatized on day 4, the protocol was adopted from Jenkins et al. (26) and Eichin et al. (27). Peritoneal cells were aseptically collected from naïve or *in vivo* stimulated mice, washed with sterile PBS, and plated 5 × 10^5^ cells/ml in six-well plates. The cells were incubated with RPMI complemented with 5% FCS, 100 μg/ml streptomycin, and 100 U/ml penicillin for 70 h in +37°C in humidified incubator with 5% CO_2_. Cells were scraped, spun down, and washed with PBS.

### Flow Cytometry

Flow cytometry was performed as previously described (3, 28), shortly Fc-receptors were blocked and the cells stained with directly conjugated antibodies. To extracellularly stain the cells CD11b (V450 Rat anti-Mouse, Clone M1/70; BD #560455), Ly6g (PE-Cy™7 Rat Anti-Mouse, Clone 1A8, BD #560601) and F4/80 (#MF48017, Invitrogen) was used. Biotinylated anti-MR (Biotin #MCA2235BT AbD Serotec) followed by Streptavidin-RPE Alexa Flour 647 (#S20992 Invitrogen) were used for intracellular staining by using BD Cytofix/Cytoperm™ (#554722, BD Bioscience) according to the manufacture’s protocol. Measured in LSR II or LSR Fortessa (BD Bioscience), analysed with FlowJo software (Tree Star, Inc.). When the expression levels of MR were evaluated, the MR-specific signal was determined as the increase in geometric mean in response to a control antibody (BD Pharmingen #555533).

### L-012 Imaging of ROS Production *In Vivo*

20 mg/kg L-012 probe (Wako Chemicals, Neuss, Germany) dissolved in physiological saline was injected i.p. into isoflurane-anesthetized mice as previously described (29). The luminescent signal was detected with the IVIS 50 bioluminescent system (Xenogen, Alameda, CA, USA), and the analyses were performed with Living Image software version 2.50 (Xenogen).

### Statistical Analysis

The graphs and statistical analyses were performed in GraphPad Prism software (GraphPad Software, Inc.), version 5.0. For comparison of two sample groups the Mann–Whitney test was utilized. *p < 0.05, **p < 0.01, and ***p < 0.001 was considered as significant.

## Results

### MR Is Protective in both the MIP and Mannan-Enhanced Rheumatoid-Like Arthritis

**Figure 1:**
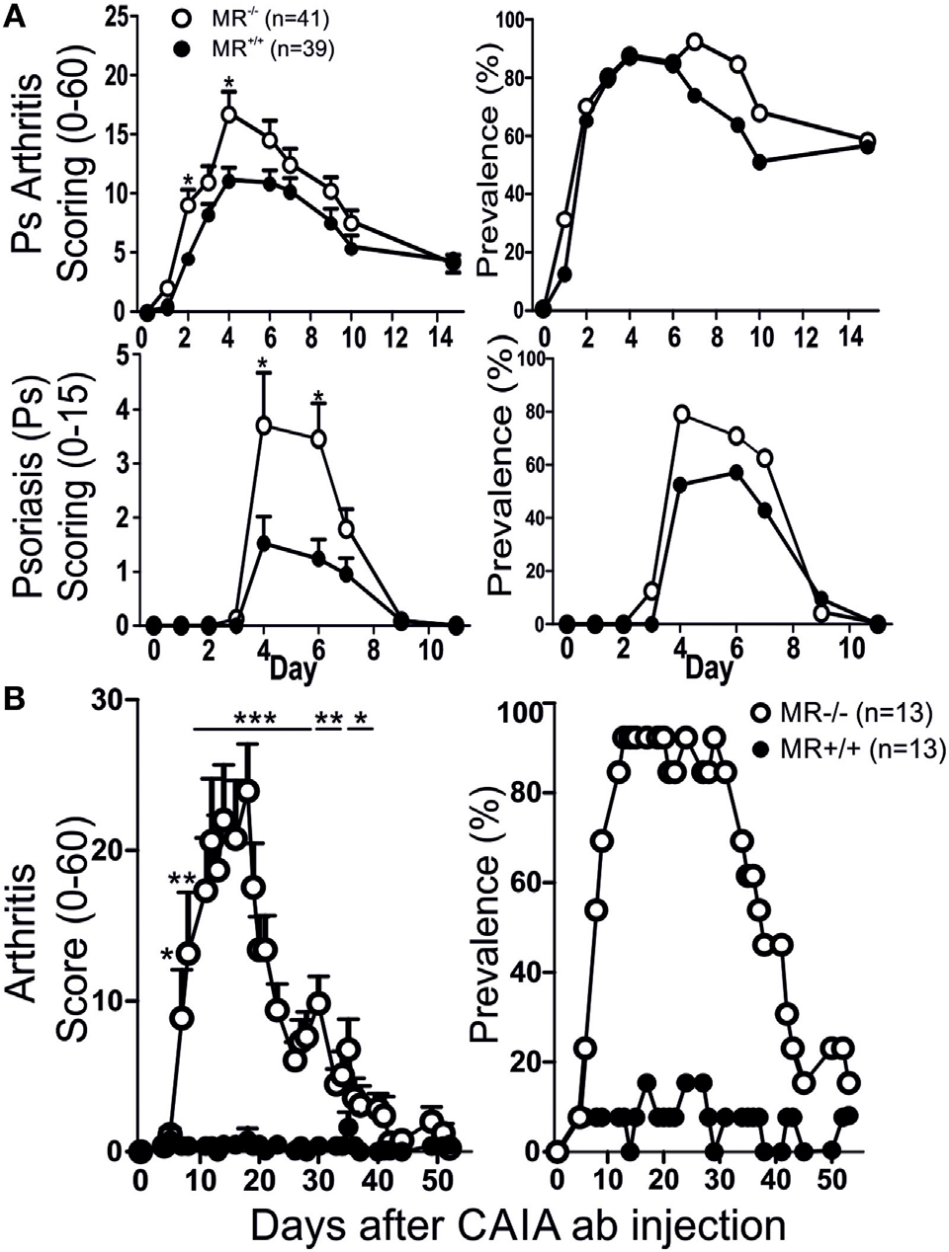
MR-deficient mice develop more severe disease. Psoriatic arthritis, psoriasis (Ps) [**(A)** two pooled experiments], and arthritis **(B)** was more severe in MR-deficient mice compared to controls, using mannan-induced psoriasis and mannan-enhanced collagen antibody-induced arthritis (CAIA), respectively. Illustrated mice are age matched non-littermates and mixed sexes. Statistical analysis performed using Mann–Whitney, *p < 0.05, **p < 0.01, ***p < 0.001. MR, macrophage mannose receptor. Values are mean ± SEM.

Mannose receptor-deficient (*MR*^−/−^) mice, with the B10.Q background, were subjected to MIP and evaluated for disease severity by comparing them both to non-littermate and littermate controls. The KO mice were found to have significantly more severe Ps and PsA disease compared to WT controls (Figure 1A). No difference was observed between littermate and non-littermate controls (Figure 1A; Figure S1 in Supplementary Material) indicating that there is no other non-linked gene influencing the results. The mice were also subjected to mCAIA, showing that loss of MR can break tolerance against the disease (Figure 1B).

### The Protective Effect by MR Is Diminished if a ROS Deficient Environment Is Introduced

**Figure 2:**
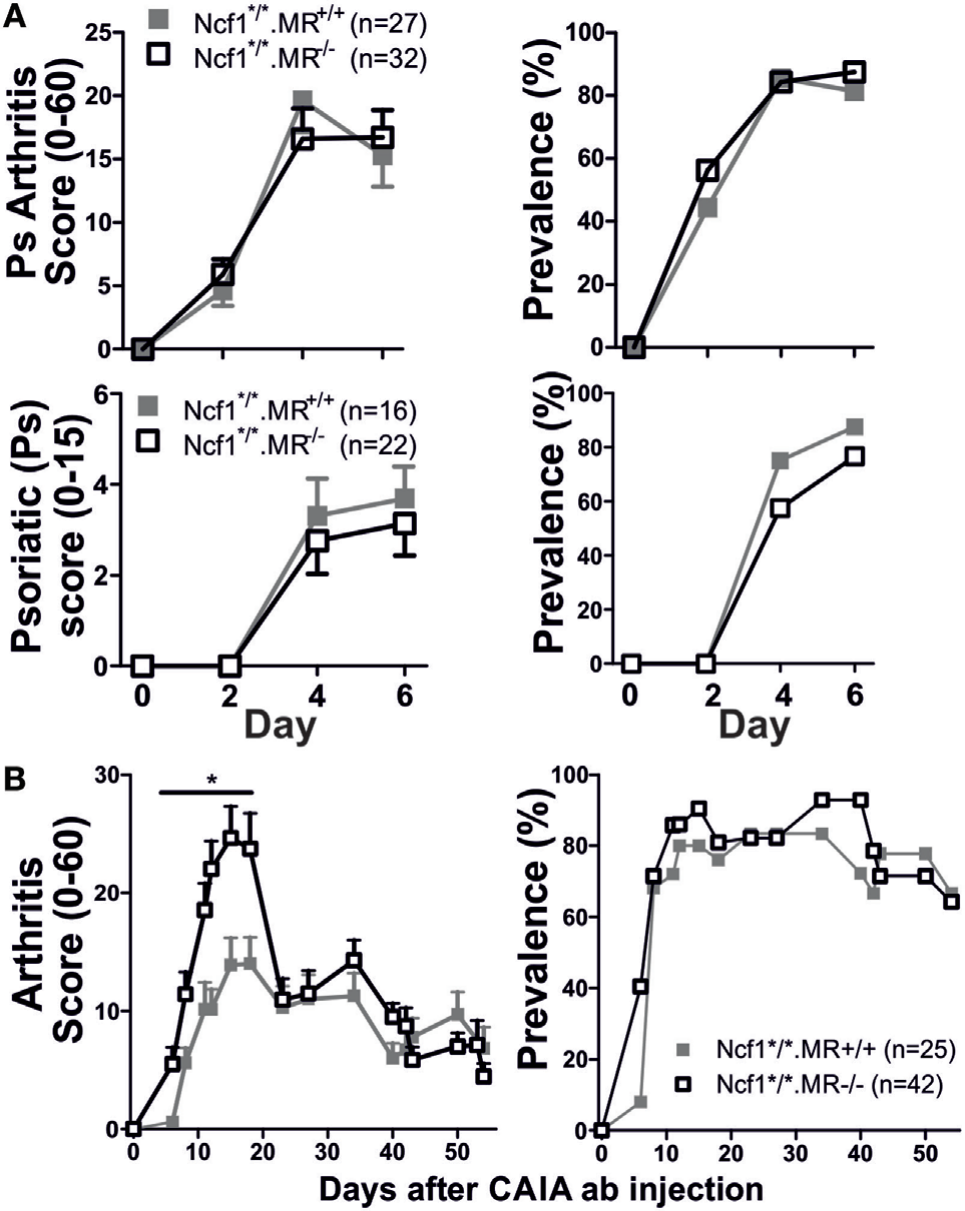
Reactive oxygen species deficient mice develop severe psoriasis (Ps) and psoriatic arthritis [**(A)** *n* = 27–32 for arthritis graphs and *n* = 16–21 for Ps graphs consisting of three and two pooled experiments, respectively] and arthritis [**(B)** *n* = 25–42, four pooled experiments]. The mannan-induced psoriasis model is no longer affected by MR, while the mannan-enhanced collagen antibody-induced arthritis (CAIA) model is **(B)**. Statistical analyses were performed using Mann–Whitney, *p < 0,05. The mice are littermates of mixed sex **(A)** or only males **(B)**. MR, macrophage mannose receptor; Ncf1^*/*^, neutrophil cytosolic factor 1 mutation m1J/m1J. Values are mean ± SEM.

Since the Ncf1^m1J^ mutation, that causes deficient ROS production, has been proven to induce a more severe disease compared to wild-type mice (3), we investigated the role of MR in a ROS deficient environment utilizing the *BQ.Ncf1^m1J/m1J^.MR^−/−^* mice. Interestingly, Ncf1^m1J^ mutated MR^−/−^ mice showed no difference in severity and prevalence of arthritis, nor psoriatic symptoms compared to *BQ.Ncf1^m1J/m1J^* (Figure 2A). However, subjected to mCAIA, the ROS and MR-deficient mice did develop more severe disease (Figure 2B), although much less pronounced than in the mice with WT Ncf1 (Figure 1B). These data indicate that MR plays a suppressive role in mannan-induced diseases and that the suppressive effect interacts with the ROS producing NOX2 complex.

### MR Interacts with NOX2-Dependent ROS and the Immune System

**Figure 3:**
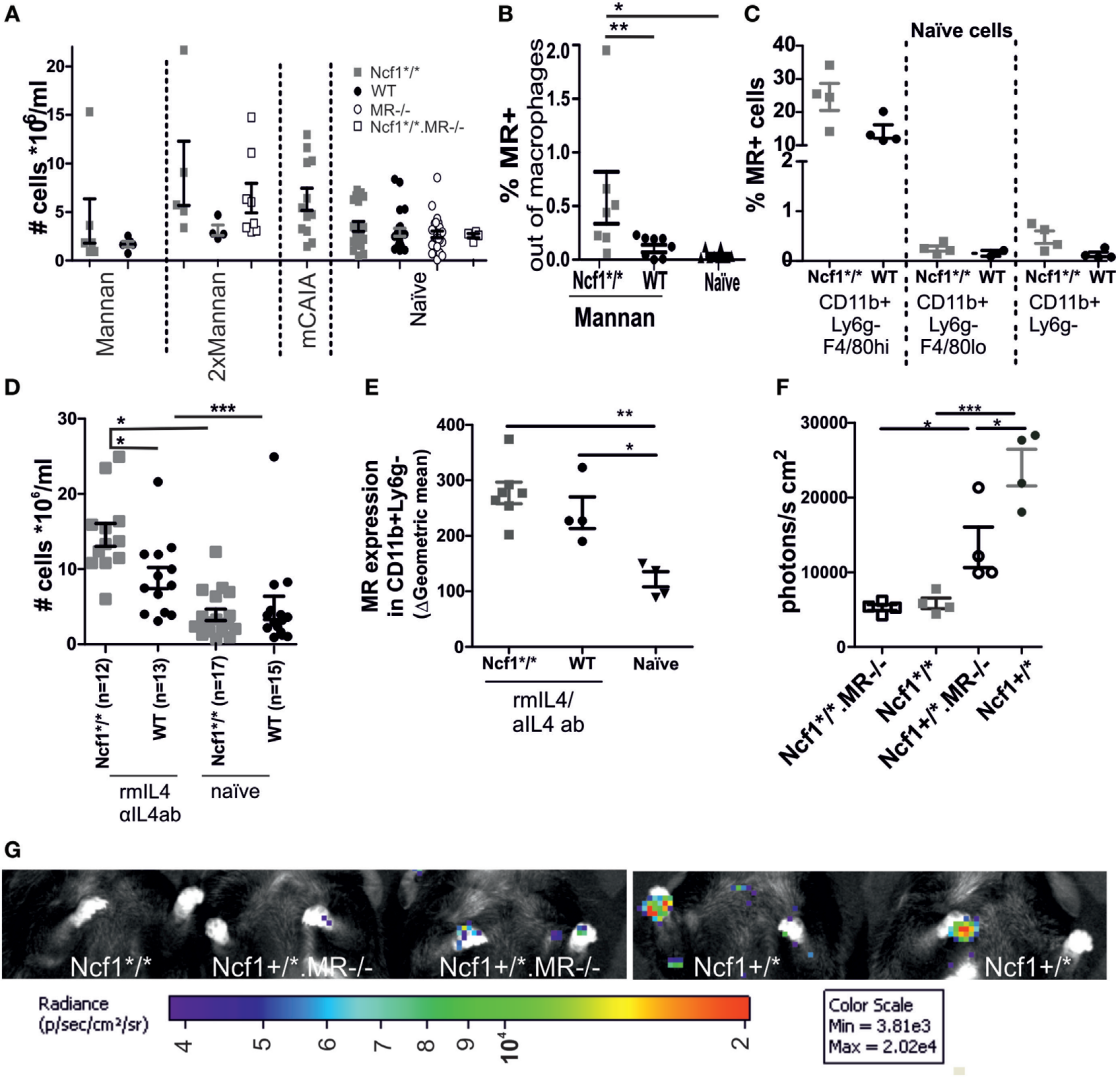
Association between MR and reactive oxygen species (ROS). **(A)** Peritoneal cell levels was measured in naïve, mannan-induced psoriasis (collected d7 p.i.), mice injected twice with mannan (d0 and d18 sample collected d24), or mannan-enhanced collagen antibody-induced arthritis (collected d95 p.i. anti-collagen type II antibody injection) treated mice (*n* = 4–21, seven combined experiments). **(B)** Peritoneal fluid collected on day 1 after the mice were subjected to i.p. injections with mannan shows an increase in MR expressing macrophages (CD68^+^ and/or F4/80^+^) in the ROS-deficient compared to the WT and naïve mice (*n* = 7–10). **(C)** MR expression in naïve peritoneal cells in CD11b^+^Ly6g^−^F4/80^hi^ (resident macrophages), CD11b^+^Ly6g^−^F4/80^lo^ (infiltrating macrophages), and CD11b^+^Ly6g^−^macrophages. **(D)** Ncf1^*/*^ and wild-type mice had an elevated number of cells in peritoneum upon *in vivo* stimulation with recombinant IL4/anti-IL4 antibody (*n* = 12–17). **(E)** i.v. injection with a combination of recombinant murine IL4 and anti-IL4 antibodies on d0 and d2, stimulates an increased expression of MR on macrophages (CD11b ^+^, Ly6G^−^) at d4 in both the Ncf1^*/*^ and the WT mice (gating strategies can be found in Figure S1 in Supplementary Material). **(F)** Lower levels of ROS are seen in MR mice after injection of mannan, as visualized by injection of L-012 1 day after mannan and subsequent imaging (*n* = 4 mean data from front paws), representative images **(G)**. MR, macrophage mannose receptor; Ncf1^*/*^, neutrophil cytosolic factor 1 mutation m1J/m1J; rmIL4, recombinant murine interleukin 4; αIL4ab, anti-interleukin 4 antibody. The data are shown as mean ± SEM. Statistical analysis Mann–Whitney, *p < 0.05, **p < 0.01, ***p < 0.001.

Both the MIP-like and the rheumatoid-like disease models are dependent on macrophage activation. As the ROS effect also operates through macrophages (30), we studied the macrophages of *Ncf1* mutant and/or *MR*-deficient mice and compared them to WT control mice. No effect on the number of peritoneal cells could be observed in *MR*-deficient or *Ncf1*-deficient mice that were subjected to mannan once, twice, or in combination with CAIA antibodies (Figure 3A). However, the number of MR positive peritoneal macrophages expanded in ROS-deficient mice compared to WT controls (Figure 3B). No difference was seen in MR expression in peritoneal macrophages from naïve *Ncf1*-deficient and WT mice (Figure 3C). To investigate whether the MR stimulation promote differentiation into M2 macrophages, peritoneal cells were stimulated twice *in vivo* with a mix of recombinant mouse IL4 and anti-IL4 antibody, using an established protocol to expand M2 macrophages *in vivo* (26, 27). As previously reported (26); rmIL4 injections increased the levels of peritoneal cells. This is even more pronounced in the ROS-deficient mice (Figure 3D), indicating an effect by ROS on M2. It was found that the ROS-deficient mice more readily upregulated MR and had a more pronounced expansion of peritoneal macrophage numbers (Figure 3E) compared to WT controls. To illustrate this effect *in vivo*, the mice were injected with the luminescent probe L-012, which upon contact with ROS increases its luminescent signal (29), showing a decrease in ROS signal in MR-deficient mice (Figures 3F,G).

## Discussion

In this work, we have shown a protective role for MR in inflammatory diseases that are dependent on the innate immune system, such as rheumatoid-like arthritis and MIP. The effect was associated with a ROS-dependent regulation of M2 macrophages. This is the first time a protective effect is seen by MR and interestingly it is regulating the innate and not the adaptive immune system.

The observation that MR operates to control innate immunity rather than the adaptive immune system came as a surprise because the main function of MR is so far believed to be involved in regulating antigen presentation and trafficking of lymphocytes to the draining lymph nodes. MR is a scavenger receptor with preference for glycosylated proteins and for collagens. It is believed that its role is to absorb glycosylated proteins from pathogens and facilitate antigen presentation of their antigens to the immune system. Mannosylated proteins and mannan are among the ligands that can be taken up. It could be hypothesized that the elevated disease in the MR KO mouse is primarily because of reduced mannan uptake, causing larger amounts of free mannan, leading to more severe disease has not been addressed in this study. However, it is unlikely that lack of proper scavenging of mannan is causing the regulation of the disease since MR are just one out of many scavenger receptors able to bind mannan. The uptake of mannan by MR is, however, likely to lead to antigen presentation and activation of the macrophages (11, 12). In the present work, we investigated inflammatory disease models for Ps, PsA, and RA that were all triggered by mannan but are also dependent on the innate immune system with no detectable involvement of the adaptive immunity (3, see text footnote 1). MR has also been widely used as a marker for M2 macrophages. M2 are characterized as immunosuppressive cells, playing a vital role in limiting and ending an inflammatory response (31–33). Furthermore, mannan has been described to bind to MR (12, 34), which indicates a role for the receptor in the development of MIP and mCAIA.

We also noted a regulative effect by ROS of the MR, indicating a shared pathway. Mannan injection led to a higher ROS response in MR sufficient mice as compared with mice lacking MR, indicating that mannan-mediated activation of MR leads to activation of the Ncf1 containing NOX2 complex. Thus, a possible explanation of the regulatory effect of MR is that it is due to an activation of NOX2 leading to a higher production of ROS that is well known to regulate chronic inflammation (35, 36). Lack of ROS also makes the presence of MR redundant in MIP as the observed effects are not as obvious in ROS-deficient mice as they are in mice with a normal ROS response. From *in vitro* studies ROS has been suggested to be of importance for differentiation of macrophages into M2 based on experiments where ROS is inhibited using butylated hydroxyanisole, and concluding that M1 cells can develop without ROS while M2 cannot (37). In MIP and mCAIA, this would mean that the disease could be driven by M1 but the downregulating M2 cells cannot be active. However, our ROS-deficient mice (Ncf1^m1J/m1J^) have M2 macrophages *in vivo*, since these have MR, arginase and YM1 expressing macrophages (data not shown). This makes it more unlikely that ROS operates through complete blocking of the differentiation toward M2 cells *in vivo*, rather ROS might affect the signaling cascade from certain receptors, such as MR, so that they become less effective. In light of the difference seen in ROS deficient mice between the MIP model and the mCAIA model one could hypothesize that this could indicate a more important role of M2 downregulating the mCAIA compared to the MIP model. In fact, the M1/M2 balance may have a role in ROS regulation of the disease models since both MIP and mCAIA are exaggerated when induced in mice with a ROS-deficient macrophages. This was previously indicated *in situ* where more similarities between the genes expressed in cells from Ps patients and IFNγ-treated cells (M1 phenotype) were observed compared to cells from Ps patients and IL4 treated cells (M2 phenotype). This together with other data indicates a driving role for the M1 cells in Ps and possibly a suppression of the M2 phenotype in the Ps patients (38). Although macrophages seem to play a vital role in both diseases it is likely that more pathways, and possibly other cell types, are affected by the ROS regulation. This is evident in the enhanced upregulation of total cell population in peritoneum in the Ncf1-deficient mice compared to control mice in IL4 treated mice, but subsequent analyzation of the MR expression fail to indicate a significant difference between the groups. For example, the STAT1/IFN I pathway and the IL17 induced pathway has been implicated to be regulated by ROS and to play a role in development of arthritis and Ps. In fact, inhibitors toward different cytokines are successfully used in Ps, PsA and arthritis. The MIP model was previously shown to have upregulated levels of IL17 and TNF. It is, however, unlikely that MR has a direct effect on these cytokines since they are not necessarily produced in the same cell type. In fact, using imiquimod induced Ps, MR was shown to be enhanced in sick mice compared to healthy, but IL17 treatment failed to reduce the MR expression (3, 28, 39–42). It is also possible that the difference seen in ROS regulation of the suppressive effect of MR seen between MIP and mCAIA could be due to differences in different roles of cytokines in these models.

Mannose receptor-deficient mice have also been shown to have a decreased adhesion of lymphocytes to the lymphatics compared to WT mice. A lower adhesion of the lymphocytes to the lymphatic system, caused by the lack of MR, should theoretically decrease the spreading of the disease: not increase it. However, Marttila-Ichihara et al. (11) have shown a reduction of lymphocyte migration from the skin into the draining lymph nodes through the afferent lymphatics in MR-deficient mice. It is not clarified, however, whether also innate lymphocytes are affected and a reduced amount of such lymphocytes leaving the skin and joints may cause increase in psoriatic and joint lesions seen in the MR-deficient mice.

Since several studies have found an enhanced expression of MR in patients with atopic dermatitis and Ps compared to normal human skin biopsies (13, 14, 38), our findings may have clinical importance. Also, Heftdal et al. (43) recently reported elevated levels of soluble MR in patients with early arthritis. Furthermore, they observed a decrease in MR levels when patients were successfully treated with anti-TNFα and DMARDs. Taken together, the upregulation of MR in human disease and our results utilizing the MR-deficient mice indicates that an activation of MR initiates a protective pathway in autoimmune diseases. Further studies are needed to conclude which receptor is important for inflammatory effect of mannan, but the observation that MR protect against Ps and arthritis in the mouse opens a possibility to investigate a new pathway and the development of novel therapies.

## Ethics Statement

This study was carried out in accordance with the recommendations of ethical permit numbers ESAVI-0000497/041003/2011 and ESAVI/439/04.10.07/2017 issued by the National Animal Experiment Board of Finland. The protocol was approved by the National Animal Experiment Board of Finland.

## Author Contributions

Acquisition of data and analysis: CH and TK. Conception or design of the work: CH, OS, TK, SJ, and RH. Drafting the manuscript: CH, OS, and RH. Revising the manuscript: TK and SJ. All authors ensure the accuracy of this work.

## Conflict of Interest Statement

The authors declare that the research was conducted in the absence of any commercial or financial relationships that could be construed as a potential conflict of interest.

## Footnotes

^1^Hagert C, Sareila O, Kelkka T, Nandakumar KS, Collin M, Xu B, et al. Chronic active arthritis driven by macrophages without involvement of T cells. *Arthritis Rheumatol* (in revision).

## Abbreviations

CAIA, collagen antibody-induced arthritis; HLA, human leukocyte antigen; mCAIA, Mannan-enhanced collagen antibody-induced arthritis; M2, type 2 macrophages; MIP, mannan-induced psoriasis; MR, mannose receptor; Ncf1, neutrophil cytosolic factor 1; rmIL4, recombinant murine interleukine 4; αIL4ab, anti-interleukine 4 antibody; Ps, psoriasis; PsA, psoriatic arthritis; PTPN22, protein tyrosine phosphatase non-receptor type 22; RA, rheumatoid arthritis; ROS, reactive oxygen species.
