# Supplementary material for: The Macrophage Mannose Receptor Regulate Mannan-Induced Psoriasis, Psoriatic Arthritis, and Rheumatoid Arthritis-Like Disease Models
Source: Front Immunol. 2018 Feb 6;9:114. doi: 10.3389/fimmu.2018.00114 (PMC5808283; doi:10.3389/fimmu.2018.00114)
Supplement: Supplementary file 1 [file presentation_1.PDF]

Supplementary Material

The macrophage mannose receptor has a protective role in both mannan induced psoriasis and arthritis

Cecilia Hagert[1,2], Outi Sareila[1,3], Tiina Kelkka[1,4], Sirpa Jalkanen[1], Rikard Holmdahl[1,3*]

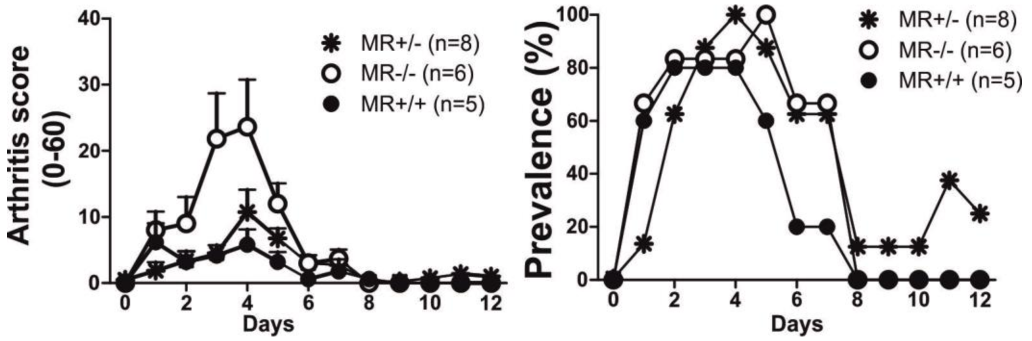

Supplement figure 1. Littermate experiment performed in backcross N3 shows similiar results as the non-littermate experiment (Figure 1A). MR; macrophage mannose receptor

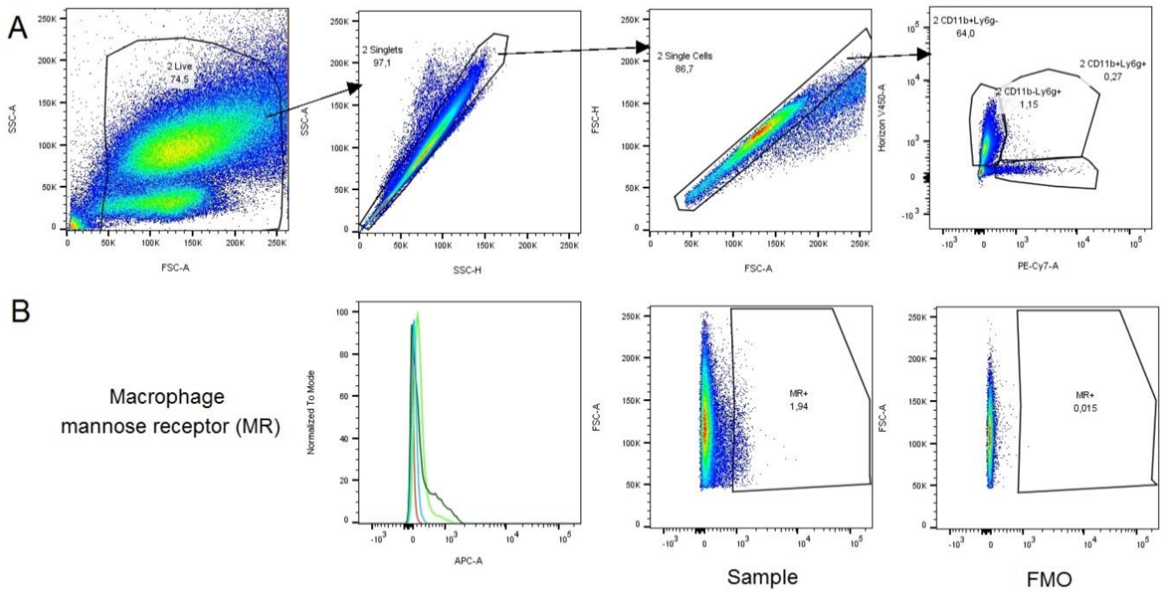

Supplement figure 2. Gating strategy for figure 3D (A). Histogram and dotplots of representative samples from the MR gating. Histogram and dotplots from representative sample and the FMO (B). In histogram red is pooled sample without any added antibodies, blue is pooled sample with all antibodies but for the one binding MR and green is a representative samples. FMO; Fluorescence Minus One.
